# *Schisandrae chinensis Fructus* Extract Ameliorates Muscle Atrophy in Streptozotocin-Induced Diabetic Mice by Downregulation of the CREB-KLF15 and Autophagy–Lysosomal Pathways

**DOI:** 10.3390/cells10092283

**Published:** 2021-09-02

**Authors:** Ho-Jung Choi, Myeong-Hoon Yeon, Hee-Sook Jun

**Affiliations:** 1 Gachon Institute of Pharmaceutical Science, College of Pharmacy, Gachon University, 191 Hambakmoe-ro, Yeonsu-gu, Incheon 21936, Korea; hchoi@gachon.ac.kr (H.-J.C.); mm257@naver.com (M.-H.Y.); 2 Lee Gil Ya Cancer and Diabetes Institute, Gachon University, 155 Gaetbeol-ro, Yeonsu-gu, Incheon 21999, Korea; 3 Gachon Medical Research Institute, Gil Hospital, 21 Namdong-daero 774 beon-gil, Namdong-gu, Incheon 21565, Korea

**Keywords:** *Schisandrae chinensis Fructus*, muscle wasting, muscle protein degradation, streptozotocin-induced diabetic mice

## Abstract

Type 1 diabetes mellitus is an autoimmune disease caused by the destruction of pancreatic beta cells. Many patients with type 1 diabetes experience skeletal muscle wasting. Although the link between type 1 diabetes and muscle wasting is not clearly known, insulin insufficiency and hyperglycemia may contribute to decreased muscle mass. In this study, we investigated the therapeutic effect of the ethanolic extract of *Schisandrae chinensis Fructus* (SFe) on muscle wasting in streptozotocin (STZ)-induced diabetic mice. STZ-diabetic C57BL/6 mice (blood glucose level ≥300 mg/dL) were orally administered SFe (250 or 500 mg/kg/day) for 6 weeks. We observed that SFe administration did not change blood glucose levels but increased gastrocnemius muscle weight, cross-sectional area, and grip strength in STZ-induced diabetic mice. Administration of SFe (500 mg/kg) decreased the expression of atrophic factors, such as MuRF1 and atrogin-1, but did not alter the expression of muscle synthetic factors. Further studies showed that SFe administration decreased the expression of KLF15 and p-CREB, which are upstream molecules of atrophic factors. Examination of the expression of molecules involved in autophagy–lysosomal pathways (e.g., p62/SQSTM1, Atg7, Beclin-1, ULK-1, LC3-I, and LC3-II) revealed that SFe administration significantly decreased the expression of p62/SQSTM1, LC3-I, and LC3-II; however, no changes were observed in the expression of Atg7, Beclin-1, or ULK-1. Our results suggest that SFe ameliorated muscle wasting in STZ-induced diabetic mice by decreasing protein degradation via downregulation of the CREB-KLF15-mediated UPS system and the p62/SQSTM1-mediated autophagy–lysosomal pathway.

## 1. Introduction

Skeletal muscle atrophy is characterized by the loss of muscle mass and muscle function and has many causes, such as aging, prolonged disuse, malnutrition, and various diseases (e.g., cancer, diabetes, and chronic kidney disease) [1,2]. Muscle atrophy adversely affects quality of life and increases morbidity and mortality [3], recovery of muscle mass and strength is essential for increasing physical performance and maintaining quality of life. Several methods have been implemented for muscle atrophy treatment. These include physical therapy such as stretching, endurance and resistance exercises to prevent immobility, functional electric stimulation for muscle contraction, or surgery in patients with muscle atrophy resulting from neurological problems [4]. However, these methods have proven largely ineffective for the treatment of muscle atrophy, leading many researchers to explore novel medication methods [5]. However, currently, there is no approved drug.

Skeletal muscle wasting is characterized by an imbalance between protein synthesis and degradation. Muscle protein degradation is mediated by proteolytic systems, such as the ubiquitin–proteasome system (UPS) [6], lysosomal autophagy [7], caspase-3 [8], and calpain [9] system. In type 1 diabetes, which results from absolute insulin deficiency, high protein turnover was observed with muscle mass reduction in the entire body [10]. The study of a healthy human body did not indicate any significant increase in muscle protein synthesis due to insulin infusion; however, it significantly decreased muscle protein degradation [11]. The study of an STZ-injected diabetic rat model demonstrated that insulin supplementation suppresses protein degradation [12]. These studies suggest that insulin deficiency may be the primary cause of muscle loss in type 1 diabetes and that increased protein degradation, rather than decreased protein synthesis in muscles, might decrease muscle mass in type 1 diabetes [11,12,13]. Moreover, an increase in the ubiquitin–proteasome pathway and the activation of the autophagy system in skeletal muscles have been reported to be involved in protein degradation in type 1 diabetes [13]. 

*Schisandrae chinensis Fructus* (SF) has been widely used in traditional medicine to treat asthma, night sweats, insomnia, dry cough, urinary disorders, involuntary ejaculation, poor memory, hyperacidity, chronic diarrhea, hepatitis, and diabetes in Korea, China, and Russia. The known pharmacological effects of SF include antioxidant, antitumor, hepatoprotective, chondroprotective, antiseptic, anti-inflammatory, antiatherosclerotic, and antidiabetic activities [14,15,16,17]. SF extract has beneficial effects on dexamethasone-, disuse-, and aging-induced muscle wasting [18,19,20]. However, no study has investigated the effect of SF extract on muscle wasting in type 1 diabetes. 

Here, we investigated the effect of the ethanolic extract of SF (SFe) on skeletal muscle mass decline and function in STZ-induced diabetic mice and its molecular mechanisms. We found that SFe inhibits muscle wasting by suppressing protein degradation through the CREB-KLF15-mediated UPS system and the p62/SQSTM1-mediated autophagy–lysosomal pathway.

## 2. Materials and Methods

### 2.1. Animals

Seven-week-old male C57BL/6 mice were purchased from OrientBio (Seongnam-si, Kyunggido, Korea) and allowed to adapt for 1 week before the study. Mice were maintained at 23 ± 1 °C with 12/12 h light/dark cycles with free access to water and a regular chow diet. All animal experiments were performed in compliance with the ethical requirements of the Laboratory Animal Research Center, College of Pharmacy, Gachon University. The experimental protocol was approved by the Gachon University Institutional Animal Care and Use Committee (GIACUC-R2019037).

### 2.2. Induction of Diabetes and Administration with SFe

STZ (Sigma-Aldrich, St. Louis, MO, USA) was intraperitoneally injected at a dose of 150 mg/kg (in 0.1 M citrate buffer, pH 4.5); the same volume of 0.1 M sodium citrate was injected into the control group (CON_Vehicle). After STZ injection, blood samples were obtained from the tail vein, and glucose levels were monitored using a glucometer (Accu-Chek^®^ Performa, Roche, Basel, Switzerland). Mice with blood glucose levels >300 mg/dL were selected and randomly assigned to the vehicle group (STZ_Vehicle), 250 mg/kg SFe group (STZ_SFe_250), and 500 mg/kg SFe group (STZ_SFe_500). SFe (70% ethanolic extract, KOC Biotech, Daejeon, Korea) was dissolved in 9% Kolliphor^®^ HS 15 (Sigma-Aldrich, St. Louis, MO, USA) and 10% DMSO (Duchefa Biochemie BV, Haarlem, Netherlands). SFe was orally administered daily (250 mg/kg or 500 mg/kg body weight) for 6 weeks. The same volume of 9% Kolliphor^®^ HS 15 + 10% DMSO was administered to the vehicle control group. Body weight, food intake, and water intake were monitored daily. The random blood glucose levels were monitored weekly. Six weeks after SFe administration, the mice were sacrificed under anesthesia and the liver, kidney, and epididymal adipose tissue were excised. Additionally, the tibialis anterior (TA), gastrocnemius (GA), and quadriceps (QD) muscles were excised from the lower limbs; wet tissue weights were measured and divided by body weight and wet tissue.

### 2.3. Grip Strength Measurement

After 40 days of SFe administration, the mice were subjected to grip strength analysis to measure muscle force. Limb grip strength was measured using a grip strength meter (BIO-G53, BIOSEB, Pinellas Park, FL, USA). To assess forelimb strength, the mice were allowed to rest on a T-bar, tightly gripping the T-bar using only the two forelimbs. The tail of each mouse was pulled directly toward the tester and parallel to the T-bar with the same force. Each mouse was analyzed in three independent replicates. Grip strength was calculated as the force divided by the final body weight (N/g).

### 2.4. Histology

GA muscles were fixed in 10% neutral buffered formalin and embedded in paraffin. The TA and QD muscles were immersed in optimal cutting temperature (OCT) solution immediately after incision and subsequently frozen at −80 °C deep freezer (Thermo-fisher scientific, Middlesex County, MA, USA). The paraffin and OCT blocks were cut into 4 µm and 10 µm thick sections, respectively, and stained with hematoxylin (30002, Muto Pure Chemicals Co., Ltd., Tokyo, Japan) and eosin (HT110132, Sigma-Aldrich, St. Louis, MO, USA) (H&E), respectively. The H&E-stained sections were used for cross-sectional area (CSA) analyses and examined (200× magnification) using a confocal microscope (Nikon Intensilight C-HGFI, Tokyo, Japan) and NIS-element AR 4.00.00 software. The myofiber cross-sectional areas were analyzed using ImageJ software (NIH, Bethesda, MD, USA).

### 2.5. Multicolor Immunofluorescent Staining

The GA muscle was immersed in OCT solution immediately after incision and frozen at −80 °C. The OCT blocks were cut to a thickness of 10 μm. The tissue was fixed in 10% neutral buffered formalin (NBF) for 20 min at RT and washed with phosphate-buffered saline (PBS). The fixed tissue was subsequently permeabilized at 25 °C in PBS containing 0.2% Triton X-100 for 40 min, before being incubated with a protein blocking solution (Dako, CA, USA) at 25 °C for 1 h. For fiber composition characterization of the skeletal muscles, tissue samples were immunolabeled with the following mouse monoclonal antibodies (Developmental Studies Hybridoma Bank (DSHB), Houston, TX, USA): anti-type I (BA-F8, mouse IgG2b), anti-type IIa (SC-71, mouse IgG1), and anti-type IIb (BF-F3, mouse IgM). The sections were incubated overnight at 4 °C in a mixture of BA-F8, SC-71, and BF-F3 antibodies (1:100), followed by incubation with secondary antibodies—DyLight 405 rabbit anti-mouse IgG2B (for BA-F8), Alexa Fluor 488-labeled goat anti-mouse IgG1 (for SC-71), and Alexa Fluor 555-labeled goat anti-mouse IgM (for BF-F3)—for 1 h at 25 °C. Fluorescent images of the cells were taken using a laser scanning confocal microscope (A1 plus, Nikon, Tokyo, Japan) at 100× magnification, and the myofiber CSA was analyzed using ImageJ software. Purple, green, and red labeled fibers were identified as type I, type IIa, and type IIb, respectively.

### 2.6. Western Blotting

The tissues were lysed using a protein extraction kit (GE Healthcare, Piscataway, NJ, USA). Protein samples (30 μg) were separated by 10% SDS-PAGE and electrophoretically transferred to a polyvinylidene difluoride membrane. The membrane was blocked by incubation in 5% bovine serum albumin for 1 h and subsequently incubated with primary antibodies against cAMP response element binding protein (CREB), phospho-CREB (p-CREB), Krüppel-like factor 15 (KLF15), muscle ring finger protein-1 (MuRF1), atrogin-1, myostatin (MSTN), p62/sequestosome1 (SQSTM1), Atg7, microtubule-associated protein light chain 3 (LC3)-I, LC3-II, Beclin-1, unc-51-like autophagy activating kinase 1 (ULK-1), phospho-ULK-1 (p-ULK-1), ubiquitin, Akt, phospho-Akt (p-Akt), Forkhead box O1 (FOXO1), phospho-FOXO1 (p-FOXO1), p70S6 kinase (p70S6K), phospho-p70S6K (p-p70S6K), or GAPDH, which was used as a loading control. The membrane was washed three times with TBST (100 mM Tris pH 7.4, 150 mM NaCl, and 0.5% Tween-20) for 10 min and incubated with horseradish peroxidase-conjugated goat anti-rabbit IgG (Santa Cruz Biotechnology, Santa Cruz, CA, USA) or horseradish peroxidase-conjugated goat anti-mouse IgG (Santa Cruz Biotechnology, Santa Cruz, CA, USA) secondary antibodies. The target complex was detected using the Chemidoc™ XRS^+^ system with Image Lab™ software (Bio-Rad, Hercules, CA, USA). The experiment was performed using five samples from each group, as indicated in the figure legends. The blots were quantified using ImageJ software.

### 2.7. Quantitative Real-Time PCR (qRT-PCR) Analysis

Total RNA from GA muscle was prepared using RNAiso reagent (Takara, Otsu, Japan) following the manufacturer’s protocol. Reverse transcription of 2 μg of total RNA was conducted using the first strand cDNA synthesis kit (Takara, Otsu, Japan) in a reaction volume of 20 μL. mRNA expression was analyzed by qRT-PCR using SYBR green reagent (Takara, Otsu, Japan). Each experiment was performed using four samples from each group, as indicated in the figure legends. The sequences of primer pairs are listed in Table 1.

### 2.8. Statistical Analysis

Data were presented as mean ± standard error (SE) or standard deviation (SD). Statistical analysis was performed using an unpaired parametric analysis of variance (ANOVA), followed by Dunnett’s multiple comparison test. Statistical significance was set at a *p* value < 0.05.

## 3. Results

### 3.1. Administration of SFe Improved Grip Strength and Reduced Muscle Weight in STZ-Induced Diabetic Mice

**Figure 1:**
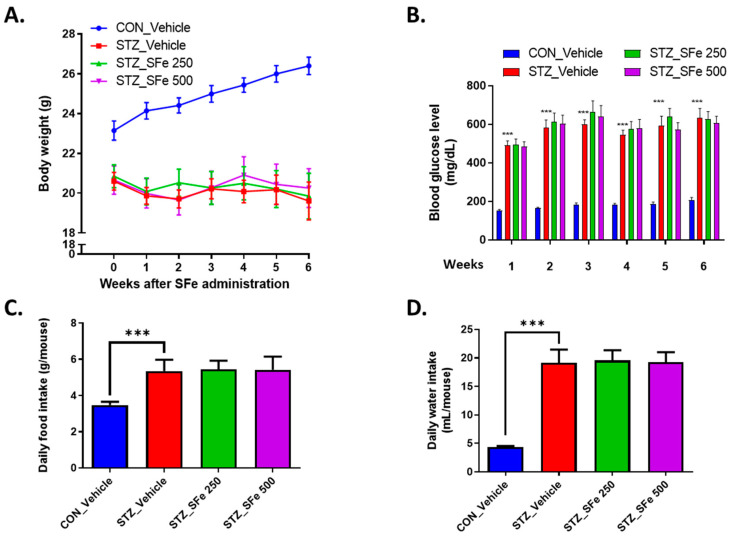
SFe administration did not affect the body weight and blood glucose levels in STZ-induced diabetic mice. Eight-week-old male C57BL/6 mice were injected with 150 mg/kg of STZ, and diabetic mice (blood glucose level >300 mg/dL) were orally administered SFe (250 or 500 mg/kg once/day) for 6 weeks. (**A**) Body weight and (**B**) non-fasting blood glucose levels were monitored weekly. (**C**) Food intake average and (**D**) water intake during the 6 weeks of SFe administration. Data are presented as the mean ± standard error (SE), n = 11 per group. *** *p* < 0.001 vs. CON_Vehicle. One-way ANOVA was performed for Dunnett’s multiple comparison test.

To evaluate whether SFe exerts beneficial effects on muscle wasting in STZ-induced diabetic mice, we administered SFe for 6 weeks to STZ-induced diabetic mice with blood glucose levels over 300 mg/dL. After STZ injection, body weight decreased; however, SFe administration did not change body weight (Figure 1A). After 6 weeks, SFe administration had not affected blood glucose levels (Figure 1B) or changed the daily food and water intake (Figure 1C,D).

**Figure 2:**
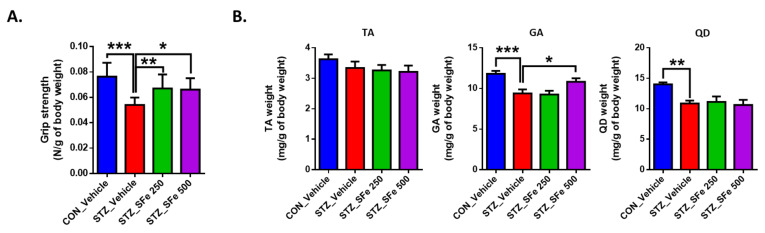
SFe administration increased the grip strength and muscle weight of GA in STZ-induced diabetic mice. (**A**) Grip strength was measured after 40 days of SFe administration. (**B**) The weights of TA, GA, and QD were measured right after sacrificing the mice and normalized to the body weight of each mouse. Data are presented as mean ± SE, n = 6 to 7/group. * *p* < 0.05, ** *p* < 0.01, or *** *p* < 0.001. One-way ANOVA was performed for Dunnett’s multiple comparison test.

We measured the grip strength to investigate the effect of SFe on muscle function in STZ-induced diabetic mice. The diabetic group (STZ_Vehicle) showed a 20% (*p* < 0.01) decrease in grip strength compared with the control group (CON_Vehicle) (Figure 2A). The administration of SFe (STZ_SFe_250 or STZ_SFe_500) significantly increased the grip strength compared with that of the STZ_Vehicle group (*p* < 0.05), with similar effects at two different doses. We also measured the weight of various muscle types including the tibialis anterior (TA), gastrocnemius (GA), and quadriceps (QD). GA and QD (but not TA) muscle weights in the STZ_Vehicle group were significantly lower than those in the CON_Vehicle group were. GA muscle weight increased after 500 mg/kg of SFe administration. However, the TA and QD muscle weights did not change (Figure 2B).

### 3.2. SFe Administration Increased Muscle Fiber Size in STZ-Induced Diabetic Mice

**Figure 3:**
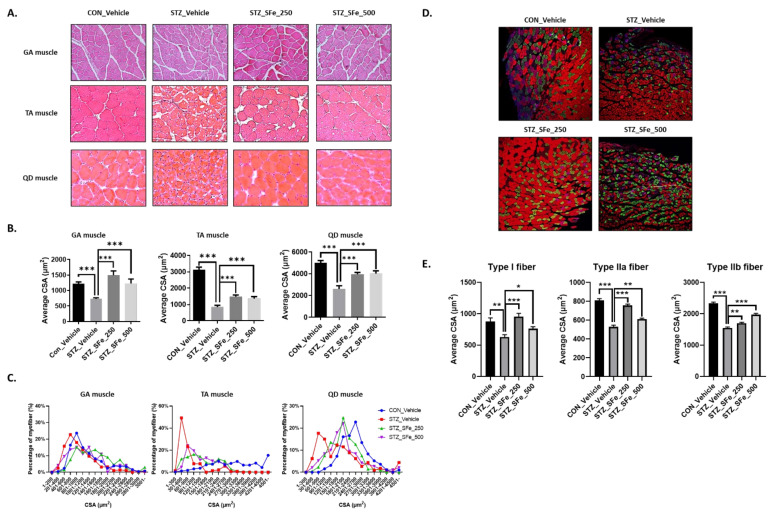
SFe administration increased the size of muscle fibers in STZ-induced diabetic mice. (**A**) H&E staining of GA, TA, and QD muscle sections. Representative images are shown (×200) (GA: n = 5 per group, TA and QD: n = 4 per group). (**B**,**C**) The cross-sectional area (CSA) distribution of muscle fibers was measured using the ImageJ program; the average CSAs (**B**) and fiber percentages (**C**) are shown (GA: n = 5 per group, TA and QD: n = 4 per group). (**D**) The multicolor fluorescent staining of GA muscle section. Representative images are shown (×100) (n = 4 per group). Purple: type I fiber, green: type IIa fiber, red: type IIb fiber. (**E**) Average CSAs of type I, IIa, and IIb fibers in GA muscle were measured using ImageJ program. Data are presented as mean ± SE. * *p* < 0.05, ** *p* < 0.01, *** *p* < 0.001. One-way ANOVA was performed for Dunnett’s multiple comparison test.

Reduced muscle function is associated with decreased muscle fiber size [21,22]. To characterize the internal structure of muscle fibers, the GA, TA, and QD muscle sections were stained with H&E, and myofiber size was analyzed. Figure 3A,B show that the approximate reductions of average CSA in GA, TA, and QD muscles by 25%, 73%, and 49%, respectively, were observed in the STZ_Vehicle group compared with the CON_Vehicle group, respectively. Administration of 250 or 500 mg/kg SFe prevented the reduction in average CSA. In STZ-induced diabetic mice, small muscle fibers are more common than large muscle fibers [23]. In the GA muscle, the predominant myofiber sizes were 801–1000, 601–800, 801–1000, and 801–1000 µm^2^ for the CON_Vehicle, STZ_Vehicle, STZ_SFe_250, and STZ_SFe_500 groups, respectively. Moreover, the percentage of myofiber sizes in the 1200–3000 µm^2^ group was higher in the STZ_SFe_250 and STZ_SFe_500 groups than in the STZ_Vehicle group in TA and QD muscles (Figure 3C). Thus, the reduced myofiber size in STZ-diabetic mice was significantly recovered by SFe administration.

As shown in Figure 2B, the wet weight of the GA muscle is higher in the STZ_SFe_500 group than in the STZ_Vehicle group. The GA muscle is a mixed muscle composed of 84.5% type IIb fibers and 17% type IIa fibers [24]. We performed multicolor fluorescent staining of type I, IIa, and IIb fibers for type-based average CSA analysis of GA muscle fibers. The average CSA of type I, IIa, and IIb fibers was lower in the STZ_Vehicle group than in the CON_Vehicle group. The administration of both 250 and 500 mg/kg SFe prevented the average CSA reduction in all fiber types, compared with the STZ_Vehicle group (Figure 3D,E). 

### 3.3. SFe Administration Decreased Protein Degradation by Inhibition of the Gene Expression of MuRF1 and Atrogin-1 in STZ-Induced Diabetic Mice

**Figure 4:**
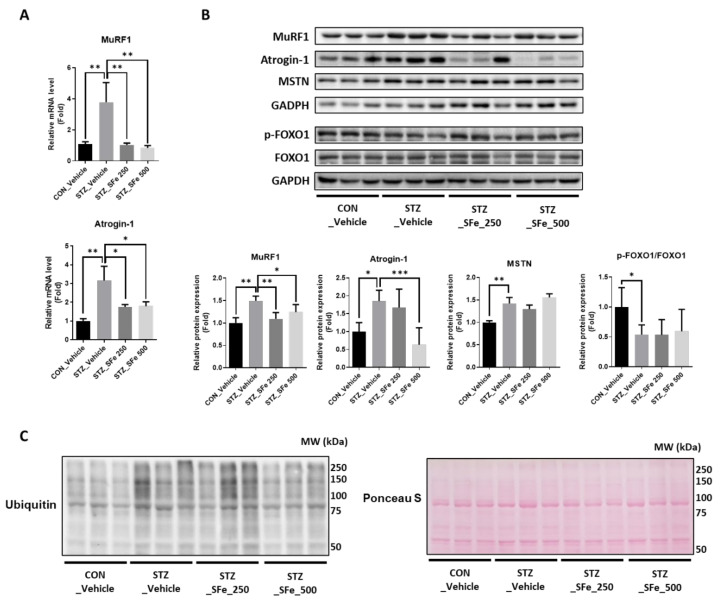
SFe administration decreased the expression of MuRF1, atrogin-1, and ubiquitin-labeled protein in STZ-induced diabetic mice. (**A**) The mRNA levels of the genes encoding MuRF1, and atrogin-1 were analyzed by RT-qPCR in GA muscle tissue. GAPDH mRNA served as an internal control (n = 4 per group). (**B**) Phospho-FOXO1, FOXO1, MuRF1, atrogin-1, and MSTN protein expression in GA muscle was analyzed by Western blotting, with GAPDH expression as an internal control. Quantitation of p-FOXO1/FOXO1, MuRF1, atrogin-1, and MSTN was computed using ImageJ software. The quantitation values are expressed as the relative fold change compared with the CON_Vehicle group. Data are presented as mean ± standard deviation (SD) (n = 5 per group). * *p* < 0.05, ** *p* < 0.01, or *** *p* < 0.001. One-way ANOVA was performed for Dunnett’s multiple comparison test. (**C**) Ubiquitin-labeled protein expression in GA muscle was analyzed by Western blotting, with Ponceau S staining used as internal control.

We examined the mRNA and protein expression levels of MuRF1 and atrogin-1, which are upregulated in STZ-induced diabetic models, to investigate the effect of SFe on the expression of muscle degradation factors in the STZ-induced diabetic model [25,26]. The mRNA expression levels of MuRF1 and atrogin-1 in GA muscle increased in the STZ_Vehicle group, and SFe administration (STZ_SFe_250 or STZ_SFe_500 group) decreased the mRNA expression of these genes (Figure 4A). In addition, protein expression levels of MuRF1 and atrogin-1 (but not myostatin (MSTN)) decreased in the STZ_SFe_250 and STZ_SFe_500 groups compared with the STZ_Vehicle group (Figure 4B). MuRF1 and atrogin-1 gene expressions are regulated by FOXO phosphorylation [27]. GA muscle of the STZ_Vehicle group exhibited decreased expression levels of p-FOXO1/FOXO1 compared to the CON_Vehicle group; however, administration of SFe did not change pFOXO1/FOXO1 levels compared with the STZ_Vehicle group (Fgure 4B). Increased E3 ubiquitin ligases, such as MuRF1 and atrogin-1, promote ubiquitin-labeled proteins. As expected, the expression of ubiquitin-labeled proteins increased significantly in the STZ_Vehicle group compared with the CON_Vehicle group, and SFe treatment (STZ_SFe_500 group) decreased the expression of ubiquitin-labeled proteins (Figure 4C). 

### 3.4. SFe Administration Did Not Affect the Expression of Molecules Related to Protein Synthesis

**Figure 5:**
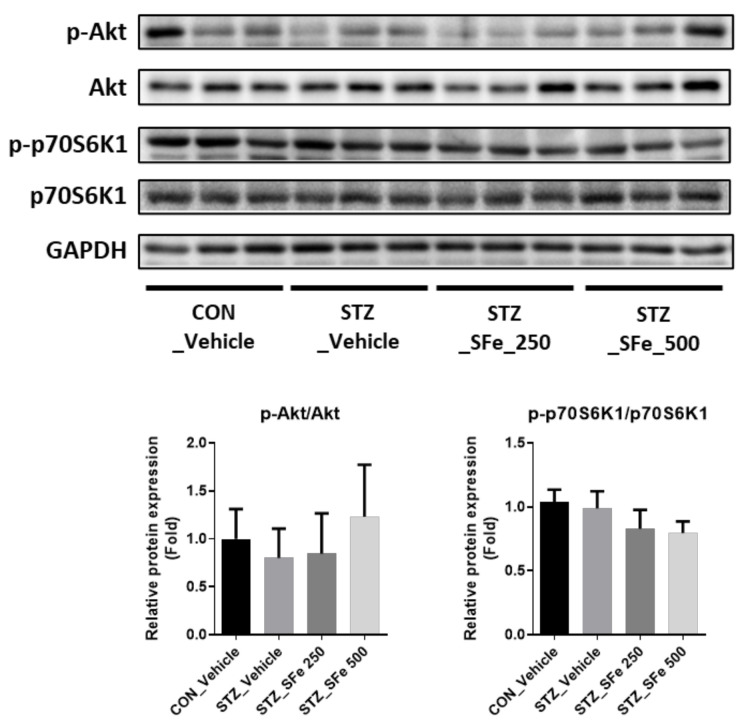
SFe administration did not affect the expression of molecules related to protein synthesis. Phospho-Akt, Akt, phospho-p70S6K1, and p70S6K1 protein expressions in GA muscle were analyzed by Western blotting; GAPDH expression was an internal control. Gene expression was quantitated using ImageJ software. Data are presented as the mean ± standard deviation (SD) (n = 5 per group).

Subsequently, we investigated whether SFe could increase the expression of molecules related to protein synthesis. Akt enhances protein synthesis by phosphorylating S6K [28]. When we checked the expression of p-Akt and p-p70S6K1, the expression levels of p-Akt/Akt or p-p70S6K1/p70S6K1 exhibited no significant differences among the experimental groups (Figure 5).

### 3.5. SFe Administration Decreased Expression of KLF15 by Inhibition of CREB Phosphorylation in STZ-Induced Diabetic Mice

**Figure 6:**
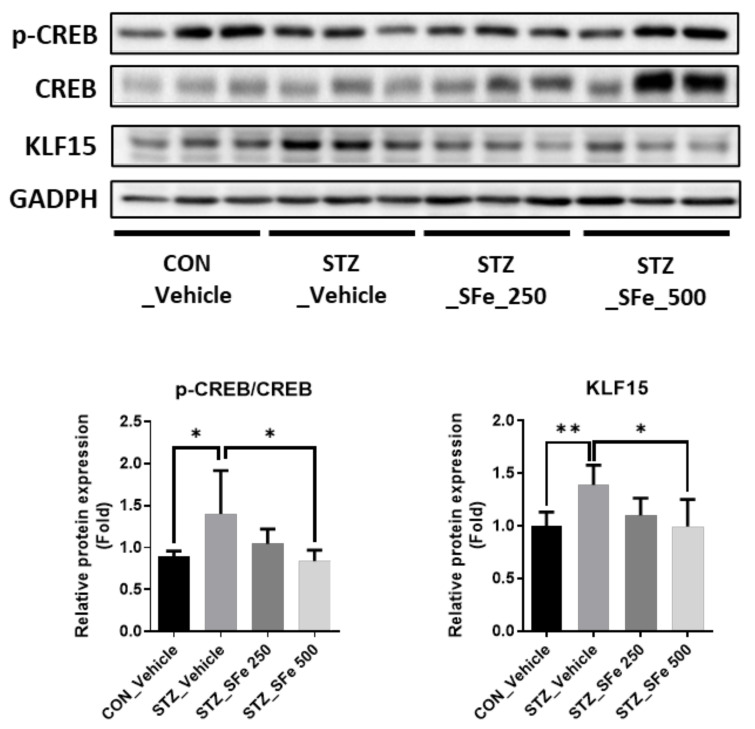
SFe administration decreased the expression of p-CREB/CREB and KLF15 in STZ-induced diabetic mice. Phospho-CREB (p-CREB), CREB, and KLF15 protein expression in GA muscle was analyzed by Western blotting, with GAPDH expression as an internal control. Quantitation of p-CREB/CREB and KLF15 was performed using ImageJ software. The quantitation values are expressed as the relative fold change compared to the CON_Vehicle group. Data are presented as the mean ± standard deviation (SD) (n = 5 per group). * *p* < 0.05 or ** *p* < 0.01. One-way ANOVA was performed for Dunnett’s multiple comparison test.

Figure 4A,B show SFe administration decreased the expression levels of two E3 ubiquitin ligases, MuRF1 and atrogin-1. To investigate the molecular mechanism, we checked the expression of KLF15, the upstream regulator of MuRF1 and atrogin-1. KLF15 is a member of the KLF family of transcription factors that regulates carbohydrate, lipid, and protein metabolism and increases MuRF1 and atrogin-1 gene transcription through crosstalk between glucocorticoid receptors [29,30,31,32]. Hyperglycemia was recently shown to promote skeletal muscle atrophy via the induction of KLF15, which is regulated by WW domain-containing E3 ubiquitin protein ligase 1 (WWP1) or cAMP-PKA/CREB signaling [33,34]. As expected, the expression of KLF15 in GA muscle increased in STZ-induced diabetic mice. Additionally, SFe administration significantly decreased the expression in the STZ_SFe_500 group and decreased CREB-mediated KLF15 protein expression levels (Figure 6).

### 3.6. SFe Administration Attenuated the Expression of Proteins Related to the Autophagy–Lysosomal Pathway in STZ-Induced Diabetic Mice

**Figure 7:**
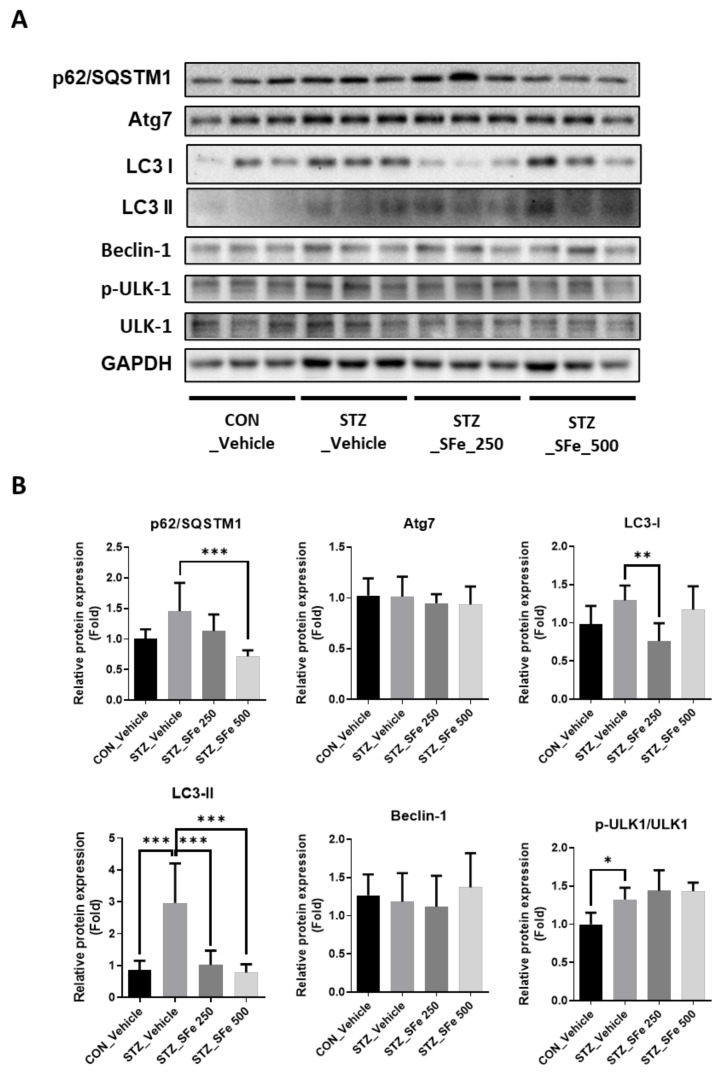
SFe decreased the expression of autophagy–lysosomal markers in STZ-induced diabetic mice. (**A**) p62/SQSTM1, Atg7, LC3-I, LC3-II, Beclin-1, p-ULK-1, and ULK-1 protein expressions in GA muscle were analyzed by Western blotting; GAPDH expression was an internal control. (**B**) Quantitation of p62/SQSTM1, Atg7, LC3-I, LC3-II, Beclin-1, and p-ULK-1/ULK-1 proteins were computed using ImageJ software. The quantitation values are expressed as the relative fold change compared with the CON_Vehicle group. Data are presented as mean ± SD (n = 5 per group). * *p* < 0.05, ** *p* < 0.01, or *** *p* < 0.001. One-way ANOVA was performed for Dunnett’s multiple comparison test.

Autophagy is a key pathway involved in protein degradation. Markers of the autophagy–lysosomal pathway (e.g., p62/SQSTM1, LC3-I, and LC3-II) were elevated following STZ injection [13,23]. p62/SQSTM1 interacts with ubiquitin-labeled proteins and autophagosomes; atg7 promotes the formation of autophagosomes; and Beclin-1 and LC3 are autophagosome components [7]. We found that SFe administration affected the expression of these proteins in STZ-induced diabetic mice. The STZ_SFe_250 group showed significantly decreased protein expression levels of LC3-I and LC3-II compared with the STZ_Vehicle group. Moreover, the STZ_SFe_500 group showed significantly decreased protein expression levels of p62/SQSTM1 and LC3-II compared with the STZ_Vehicle group (Figure 7A,B). However, the expressions of Atg7, Beclin-1, and p-ULK1/ULK1 were not significantly altered by SFe administration (Figure 7A,B).

## 4. Discussion

Muscle wasting is caused by several factors, including diabetes, aging, and various diseases [35], and is a serious health problem worldwide [36]. In particular, patients with type 1 diabetes have decreased muscle size and strength, which contribute to poor physical fitness and increased risk of disability [37,38]. In this study, we used STZ-induced diabetic mice as a type 1 diabetes animal model. STZ-induced diabetes rapidly increases blood glucose levels and causes muscle wasting syndrome, similar to human type 1 diabetes [39].

We investigated the effect of SFe on muscle wasting in the STZ-induced diabetic mouse STZ_SFe_500 group compared with the STZ_Vehicle group; however, no changes were found in the TA and QD muscles. GA and QD muscles have a higher proportion of type IIb fibers and fast twitch fibers than the TA muscle in C57BL/6 mice [24], and the GA muscle has a higher proportion of type IIb and type IIx fibers than QD muscle in CD-1 mice [40]. It was reported that STZ decreases fast fibers and promotes fast-to-slow muscle shifting [41,42,43]. We also observed the decrease in the wet weight of GA and QD muscles, which primarily contain fast fibers, in this study (Figure 2B). Similar to a previous report [44], we found that the STZ_Vehicle group exhibited a decrease in average CSA of type I, IIa, and IIb fibers compared with the CON_Vehicle group in the GA muscle, and the decrease in average CSA of type II fibers (type IIa and IIb) in the STZ_Vehicle group was higher than that of type I fibers. It was previously reported that SFe increases the percentage of slow fibers and induces the expression of myosin heavy chain 1 mRNA in the TA muscle of aged mice [20]. Similarly, our data showed that the administration of SFe strongly inhibited STZ-induced degradation in GA muscle slow fibers, compared with fast fibers (Figure 3E). Grip strength and muscle fiber size have a positive correlation [21,22]. Our results were consistent and found improved grip strength and increased GA muscle fiber size in the STZ_SFe_250 and STZ_SFe_500 groups compared with the STZ_Vehicle group. Although the size of the GA muscle fibers increased, the weight of the GA muscle in the STZ_SFe_250 group was not significantly increased compared with that in the STZ_Vehicle group. Therefore, 250 mg/kg of SFe administration may not be sufficient to increase GA muscle weight. 

An increase in blood glucose levels due to insulin deprivation (hyperglycemia) can regulate the expression of genes related to protein degradation and synthesis [23,26,33]. The increased expression of MuRF1 and atrogin-1 (protein degradation markers) in diabetic mice was downregulated at both the mRNA and protein levels by SFe treatment; however, MSTN expression did not change. These effects were more pronounced at a dose of 500 mg/kg than at 250 mg/kg. Insulin deprivation promotes protein degradation and inhibits protein synthesis in muscles [45,46]. Therefore, we also checked the expression of protein synthesis markers, such as Akt and p70S6K1 phosphorylation. We found no changes in the expression of these molecules between the STZ_Vehicle and STZ_SFe groups. Muscle protein degradation is promoted by the UPS, caspase system pathway, and autophagy–lysosomal pathways [13,47,48,49]. UPS is a key pathway that contributes to muscle loss [6]. Ubiquitin binds to the substrate of targeted proteins via the ubiquitin-activating enzyme (E1), ubiquitin-conjugating enzyme (E2), and ubiquitin ligase enzymes (E3), which activate the proteolytic signaling pathway [13]. Among them, E3 ubiquitin ligases have substrate specificity; the gene expressions of atrogin-1 and MuRF1 in the skeletal muscle increase during the atrophy process [47,48]. Arogin-1 and MuRF1 gene expression is regulated by several transcription factors, such as FOXO1 and KLF15. In this study, SFe treatment did not affect the phosphorylation of FOXO1 (Figure 4B). In hyperglycemic conditions, KLF15 protein expression increases [33], and KLF15 is regulated by CREB signaling [34]. In this study, we found that SFe treatment decreased the expression of KLF15 and p-CREB in muscles, suggesting that SFe could inhibit protein degradation by regulating MuRF1 and atrogin-1 through cAMP-PKA/CREB-KLF15 signaling.

In addition, the autophagy–lysosomal pathway is a key pathway involved in muscle protein degradation [13,49] that is induced by STZ-induced diabetic conditions [13,23]. Serval proteins, such as autophagy-related genes p62/SQSTM1 and LC3, are involved in the autophagy–lysosomal pathway under hyperglycemic conditions [13]. p62/SQSTM1 is a multifunctional protein that delivers ubiquitinated proteins to the proteasome for degradation and to activate autophagy through phosphorylation [50]. LC3 exists in two known forms. LC3-I is found in the cytoplasm, and LC3-II is membrane-bound and converted from LC3-I to initiate formation and lengthening of the autophagosome [51]. In diabetic conditions, macroautophagy is activated, and the autophagy protein LC3 accumulates in vesicles [52]. We observed that the STZ_SFe_500 group had a lower expression level of ubiquitin-labeled proteins than the STZ_Vehicle group. We also observed a decrease in p62/SQSTM1 and LC3-II protein expression levels in the STZ_SFe_500 group. Therefore, SFe decreased protein degradation by inhibiting the autophagy–lysosomal pathway in STZ-induced diabetic mice. 

SF was recently revealed to be effective in improving QD muscle strength in clinical trials [53]. SFe contains various bioactive components, including lignans, volatile oils, and fatty acids [16]. However, the bioactive components of *Schisandrae* responsible for muscle strength improvement remain undetermined, with the exception of schisandrin A [21]. Therefore, further investigation of muscle strength improvement, using several lignans including schisandrin A, will be required. 

## 5. Conclusions

Overall, we demonstrated that SFe increases muscle strength and muscle fiber size, which were reduced in STZ-induced diabetic mice showing muscle wasting. At the molecular level, SFe administration prevented the development of atrophic changes in STZ mice, which was accompanied by suppression of the CREB-KLF15-UPS and p62/SQSTM1-mediated autophagy signaling pathways. These results suggest that SFe is a potential treatment agent for muscle wasting in patients with type 1 diabetes. 

## Figures and Tables

**Table 1:** Primer sets used for quantitative PCR analyses.

No.	Primer	Sense	Antisense
1	MuRF1	AGGACTCCTGCAGAGTGACCAA	TTCTCGTCCAGGATGGCGTA
2	Atrogin-1	GCAAACACTGCCACATTCTCTC	CTTGAGGGGAAAGTGAGACG
3	18s rRNA	CCATCCAATCGGTAGTAGCG	GTAACCCGTTGAACCCCATT

## Data Availability

The data used to support the findings of this study are available from the corresponding author upon request.
